# Development of the Stanford Social Dimensions Scale: initial validation in autism spectrum disorder and in neurotypicals

**DOI:** 10.1186/s13229-019-0298-9

**Published:** 2019-12-18

**Authors:** Jennifer M. Phillips, Mirko Uljarević, Rachel K. Schuck, Salena Schapp, Elizabeth M. Solomon, Emma Salzman, Lauren Allerhand, Robin A. Libove, Thomas W. Frazier, Antonio Y. Hardan

**Affiliations:** 1 Department of Psychiatry and Behavioral Sciences, Stanford University, Stanford, CA USA; 2 Department of Psychiatry, Kaiser Permanente, Redwood City, CA USA; 3 Department of Psychiatry and Behavioral Sciences, University of California, Davis Medical Center, Sacramento, CA USA; 4 Department of Psychiatry, University of California San Francisco, San Francisco, CA USA; 5 Autism Speaks, New York, USA

**Keywords:** Autism spectrum disorder, Social processing, Social motivation

## Abstract

**Background:**

The aim of this paper was to provide an initial validation of a newly developed parent questionnaire—the Stanford Social Dimensions Scale (SSDS), designed to capture individual differences across several key social dimensions including social motivation in children and adolescents with and without psychiatric disorders.

**Methods:**

The initial validation sample was comprised of parents of 175 individuals with autism spectrum disorder (ASD) (35 females, 140 males; *M*_age_ = 7.19 years, *SD*_age_ = 3.96) and the replication sample consisted of 624 parents of children who were either typically developing or presented with a range of neurodevelopmental and neuropsychiatric disorders (302 females, 322 males; *M*_age_ = 11.49 years, SD_age_ = 4.48). Parents from both samples completed the SSDS and the Social Responsiveness Scale (SRS-2).

**Results:**

Exploratory Structural Equation Modeling indicated that a 5-factor model provided adequate to excellent fit to the data in the initial ASD sample (comparative fit index [CFI] = .940, Tucker-Lewis Index [TLI] = .919, root mean square error of approximation [RMSEA] = .048, standardized root mean square residual [SRMR] = .038). The identified factors were interpreted as Social Motivation, Social Affiliation, Expressive Social Communication, Social Recognition, and Unusual Approach. This factor structure was further confirmed in Sample 2 (CFI = 946, TLI = .930, RMSEA = .044, SRMR = .026). Internal consistency for all subscales was in the good to excellent range across both samples as indicated by Composite Reliability scores of ≥ .72. Convergent and divergent validity was strong as indexed by the pattern of correlations with relevant SRS-2 and Child Behavior Checklist domains and with verbal and non-verbal intellectual functioning scores in Sample 1 and with the Need to Belong Scale and Child Social Preference Scale scores in Sample 2. Across both samples, females had higher social motivation and expressive social communication scores. Discriminant validity was strong given that across all SSDS subscales, the ASD sample had significantly higher impairment than both the typically developing group and the group with other clinical conditions, which in turn, had significantly higher impairment than the typically developing group.

**Conclusions:**

Our findings provide initial validation of a new scale designed to comprehensively capture individual differences in social motivation and other key social dimensions in ASD.

## Background

Impairments in social functioning have been considered a defining feature of autism spectrum disorder (ASD) from the original clinical descriptions by Kanner [1] to the latest iterations of the diagnostic nomenclature [2]. Given their pervasive impact across all aspects of functioning [3, 4], social impairments constitute a primary intervention target [5, 6]. Social motivation theory suggests that during early development, children with ASD experience lower levels of social motivation, defined as the drive or desire to interact socially and affiliate with others, independent of the quality of the interaction or overture, and are consequently less likely to orient to, be exposed to, and learn from socially relevant stimuli. Although it has been suggested that reduced social motivation might negatively impacts the development and specialization of brain circuits subserving social information processing and potentially result in the impairments in social interaction and communication that characterize ASD [7, 8], dedicated longitudinal studies are needed to establish the causality and directionality of the proposed effect. In addition to providing a useful framework for understanding the emergence of social impairments in ASD, this theory has had an important impact in highlighting deficits in social motivation as a potentially important target for treatment. Indeed, children with ASD who receive interventions aimed at increasing social motivation, such as the Early Start Denver Model (ESDM) [9] or Pivotal Response Treatment (PRT) [10], have been shown to have better outcomes and require fewer services later in life [11]. In addition, individual differences in social interest and drive for social engagement occur across normative development [12] and a range of other disorders including Williams Syndrome and Schizophrenia [13, 14] and have been shown to be related to a range of outcomes across both normative and atypical development. Finally, social motivation is recognized as an important component of the Affiliation and Attachment construct described by the National Institute of Mental Health’s Research Domain Criteria (RDoC) [15]. However, despite the noted prominence of the construct, there is a paucity of instruments specifically designed to capture individual differences in social motivation.

A wide range of existing behavioral, experimental, and neuroimaging findings provide support for the social motivation theory. For instance, lack of orienting to social stimuli across both auditory [16–18] and visual [19] modalities represents one of the earliest features of ASD. These impairments continue through childhood and adolescence as evidenced by a range of eye-tracking studies which demonstrate that when compared to controls, individuals with ASD show reduced preference for social over non-social stimuli [20–24]. Neuroimaging evidence thus far suggests structural and functional atypicalities in brain regions involved in reward processing, including the nucleus accumbens, caudate, anterior cingulate cortex, ventromedial prefrontal cortex, orbitofrontal cortex, insula, amygdala, and putamen [25–27]. Functional neuroimaging studies focusing on social motivation most often employ tasks contrasting brain activation to social and non-social rewards and these studies have suggested atypical activity within the reward circuitry [28–31], although it appears that atypical processing of social rewards can be attributed to a more general deficit in the reward system [32]. Eye-tracking and neuroimaging studies provide important insights into the mechanisms behind impaired social motivation in ASD; however, apart from eye-tracking, these methods often lack ecological validity and are often not suitable for individuals with ASD who have co-occurring intellectual disability, thus necessarily limiting the generalizability of findings.

Currently available observational, interview- and questionnaire-based measures designed to diagnose and/or screen for the presence of ASD, such as the Autism Diagnostic Observation Schedule, Second Edition (ADOS-2) [33], the Autism Diagnostic Interview—Revised (ADI-R) [34], the Developmental Diagnostic Dimensional Interview (3Di) [35], and the Social Communication Questionnaire (SCQ) [36], do not directly measure social motivation. Importantly, these diagnostic and screening instruments were specifically designed to ascertain the presence of behaviors considered most indicative of ASD, and therefore, by virtue of their design, these instruments are not sensitive to subtle symptom expression and change. Dimensional measures such as the Social Responsiveness Scale, Second Edition (SRS-2) [37], the Broader Phenotype Autism Symptoms Scale (BPASS) [38], and the Autism Spectrum Quotient (AQ) [39] are sensitive to milder symptom expression and provide some coverage of social motivation, however, the number of items sampling social motivation is limited and these measures do not assess all social domains. For example, the AQ has only five items that tap into social motivation (e.g., “I prefer to do things with others rather than on my own”). In addition, these items are combined with a range of other items assessing constructs such as social cognition and social skills into an overall social skills scale. Unlike the AQ, the BPASS provides a separate score for social motivation; however, this score is limited to a total of two social motivation items (sociability with peers and sociability with groups). Originally, the SRS-2 was conceptualized to provide a unitary score across a range of social and communication impairments; however, factor analysis by Frazier et al. [40] identified five factors (emotion recognition, social avoidance, interpersonal relatedness, repetitive motor mannerisms, and insistence on sameness). Although the social avoidance factor includes several items directly related to social motivation construct (“Would rather be alone than with others” and “Avoids starting social interactions with peers or adults”), it also includes items that do not readily map onto social motivation (e.g., “Expressions on his/her face don’t match what he/she is saying”, and “Is too tense in social situations”). Finally, the Social Pleasure Scale [41] and the Social Anhedonia Scale [42] assess pleasure derived from social interactions; however, they are self-report measures and are limited in capturing social motivation in young children and individuals who are not able to self-report. Given the noted limitations of the currently existing instruments, our main aim was to develop a parent/caregiver report questionnaire that would enable a comprehensive and sensitive depiction of individual variation in social motivation, defined as the drive or desire to interact socially and affiliate with others, independent of the quality of the interaction or overture. By providing a detailed assessment of social motivation, the newly developed scale would therefore address important limitations of the current instruments.

As is the case with other core and co-occurring symptoms, there is pronounced variability in social motivation among individuals with ASD, ranging from individuals who lack social interest and awareness of others, to those who show the desire to have friendships and romantic relationships and report increased levels of loneliness [43–47]. In light of the noted heterogeneity of the social motivation domain, it is clear that interventions aimed at increasing social motivation might not be effective or even needed for all individuals with ASD. It is therefore essential to be able to effectively capture individual differences in social motivation.

Social motivation is an important element of social functioning; however, the ability to function across different social settings relies on a range of other social processes. Although comprehensive taxonomy of social functioning has not been reached yet, and considerable debate exists in terms of exact processes that constitute social functioning domain, it has been widely acknowledged that (1) the ability to perceive and interpret social signals and (2) skills necessary for initiating, maintaining, and ending social interactions are key domains and skills necessary for successful social functioning [48–51]. In addition to varying in their levels of social motivation, there are pronounced individual differences between individuals with ASD and other neurodevelopmental disorders in terms of their social recognition and expressive social communication skills and abilities. Importantly, a recent study by Livingstone et al. [52] has demonstrated that individual differences along social recognition and social communication can be used to identify potentially informative subgroups of individuals with ASD. Therefore, it is crucial for instruments to be able to capture individual’s strengths and weaknesses across these distinct components of the social phenotype. This approach is consistent with the RDoC initiative which emphasizes the importance of considering a set of basic, biologically meaningful dimensions in order to deconstruct sources of variation in social impairments across affected individuals. Although several existing measures, in particular SRS-2, provide comprehensive assessment of the expressive social communication abilities in ASD, a number of issues might limit their utility in mapping distinct social domains, in particular social recognition. For instance, although originally proposed Social Awareness and Social Cognition SRS subscales capture certain aspects of social recognition/social cognition, factorial work was not able to empirically validate these theoretically derived subscales. Recent factor analysis by Frazier et al. [40] has indicated existence of Emotion Recognition factor (in addition to 4 other factors); however, Frazier and colleagues also reported very high correlations among derived factors raising questions about their distinctiveness. Both original Social Awareness and Cognition, and empirically derived Emotion Recognition SRS-2 subscales contain a number of items that do not directly relate to the social recognition/cognition construct (for instance “Seems self-confident when interacting with others” and “Clings to others” in case on Emotion Recognition factor) further limiting their potential utility. Further, our recent work [53] has demonstrated that SCQ, another widely used measure of social impairments, does not provide coverage of the social recognition abilities. Consequently, our aim in the present investigation was to develop a set of items that capture, in addition to motivation and affiliation, social recognition and expressive social communication domains. By capturing these additional constructs, when used as a stand-alone instrument, newly developed measure would enable relatively comprehensive characterization of the social phenotype across ASD and other disorders, but would ideally be supplemented by instruments such as SRS-2.

The aim of this investigation was to provide initial validation of a newly developed instrument—the Stanford Social Dimensions Scale (SSDS). We first present data on the parental feedback and readability of the measure. Secondly, we present an initial exploration of the factorial structure of the questionnaire in an ASD sample. We examine the reliability and the association between derived factors and the global social processing domain, as indexed by the SRS-2; explore the association with externalizing and internalizing symptoms and impairments in self-regulation, as indexed by the Child Behavior Checklist (CBCL) [54]; and investigate the association with verbal and non-verbal cognitive ability. It was hypothesized that all SSDS factors would be associated more highly with the SRS-2 social communication/interaction scale than with the SRS-2 restricted/repetitive behavior scale. Further, we hypothesized that the social interest/drive and affiliation components of the instrument would be more highly associated with the CBCL internalizing scale than the externalizing scale, and that, conversely, expressive social communication and social recognition components of the instrument would be more highly associated with the CBCL externalizing than internalizing scale. Thirdly, we aimed to confirm the structure derived in our ASD sample in a larger online sample spanning typical and atypical development. Given that the primary focus of the SSDS is on social motivation, we included two dedicated measures tapping into affiliative and (lack of) social interest/drive components—the Need to Belong Scale (NTBS) [55] and the Child Social Preference Scale (CSPS) [12], respectively. It was hypothesized that the affiliation component of the SSDS would be more highly associated with the NTBS and that the interest/drive SSDS component would be more highly associated with the CSPS. In addition, we expected that the NTBS and CSPS scales would be more highly associated with the SSDS social motivation components than with the SRS-2 factor that measures this construct (social avoidance scale [40]).

## Methods

### Participants

#### Sample 1

One hundred seventy-five individuals with ASD and their parents/caregivers took part in the study (35 females, 140 males; *M*_age_ = 7.19 years, SD_age_ = 3.96, range 2–17). Participants were recruited through (1) ongoing research projects conducted in the Stanford Autism and Developmental Disabilities Research Program, (2) the Stanford Autism and Developmental Disorders Research Registry, (3) flyers posted in the Stanford Child Psychiatry Clinics, (4) advertisements posted online (e.g., parent listservs), and (5) flyers distributed at special events (e.g., Stanford Autism Center annual conference). Participants recruited through the Stanford Autism and Development Disabilities Research Program received cognitive testing using the Stanford Binet, Fifth Edition [56] and confirmatory diagnostic assessment with the ADI-R [34] and/or the ADOS-2 [33]. ADI-R and ADOS-2 were administered by research staff trained and supervised by a research-reliable clinician. For participants recruited online, inclusion criteria was a reported diagnosis of ASD and an SRS-2 total T score of 60 or greater [37, 57]. See Table 1 for the descriptive statistics of the sample.

**Table 1:** Participant characteristics

	M (SD)	Ethnicity	%	Income	%
Sample 1					
Age (years)	7.19 (3.96)	Caucasian	41.9	> 150,000	50.7
NVIQ	80.62 (26.49)	Asian	30.2	125,000–150,000	13.2
VIQ	70.02 (30.42)	Mixed Race	15.1	100,000–125,000	6.9
SRS-2 Total	76.20 (10.68)	Hispanic	8.7	75,000–100,000	10.4
CBCL Internalizing	62.39 (10.72)	Middle Eastern	1.2	50,000–75,000	8.3
CBCL Externalizing	56.50 (10.49)	Native American	1.2	35,000–50,000	4.2
CBCL-DP	183.49 (23.06)	Pacific Islander	1.2	25,000–35,000	1.4
		African American	0.6	< 25,000	4.9
Sample 2					
Age (years)	11.49 (4.48)	Caucasian	76	> 150,000	9.5
SRS-2 Total	55.07 (13.21)	Asian	5.8	125,000–150,000	5.3
SDQ Total	9.75 (7.89)	Mixed Race	6.6	100,000–125,000	11.1
NTBS	33.90 (4.76)	Hispanic	1.4	75,000–100,000	16.2
CSPS Unsociability	11.18 (3.13)	Middle Eastern	0.2	50,000–75,000	17.4
		Native American	2.1	35,000–50,000	12.2
		Pacific Islander		25,000–35,000	13.3
		African American	8.0	< 25,000	15

*CBCL* Child Behavior Checklist, *CSPS* Child Social Preferences Scale, *DP* dysregulated profile, *NVIQ* non-verbal IQ, *NTBS* Need to Belong Scale, *SDQ* Strengths and Difficulties Questionnaire, *SRS-2* Social Responsiveness Scale, *VIQ* verbal IQ

#### Sample 2

Six hundred twenty-four parents of individuals aged 2–17 years took part in this online investigation (302 females, 322 males, *M*_age_ = 11.49 years, SD_age_ = 4.48). Four hundred thirty-seven children were typically developing, and for 187 children parents reported a clinical diagnosis (*N* = 81 ADHD, *N* = 39 internalizing disorders, *N* = 32 ASD, *N* = 20 Language Delay, *N* = 7 Intellectual/Learning Disability, *N* = 5 Social/Pragmatic Communication Disorder, *N* = 2 Oppositional Defiant Disorder, *N* = 1 Sleep Disorder). Inclusion criteria for TD children were that they had a *T* score of 59 or lower on the SRS-2. Inclusion criteria for ASD was an SRS-2 *T* score of 60 or greater, and for other clinical diagnoses inclusion criteria was that they met the Strengths and Difficulties Questionnaire (SDQ) [58] total score cut-off or the cut-off score on the corresponding subscale of the SDQ (e.g., the emotional symptoms subscale for internalizing disorders or the hyperactivity/inattention symptoms subscale for ADHD). See Table 1 for the descriptive statistics of the sample and Additional file 1: Table S1 for the descriptive statistics broken down for diagnostic groups.

### Procedures and measures

#### Measures

Sample 1

*The Stanford Social Dimensions Scale (SSDS).* An initial set of items was conceptually developed by the authors (JMP, AYH, SS, EMS, ES) after a comprehensive literature review, and through consultation with clinicians and experts in the field of ASD, in order to tap into the social interest/drive and affiliation components of social motivation, as well as the constructs of expressive social communication and social recognition described above. The initial list of items was developed to reflect the full range of behaviors seen in normative social development and ASD as well as across neurodevelopmental disorders. The final items were retained based on consensus among the authors based on the appraised relevance of each item for each of the constructs. The preliminary version of the instrument contained 58 items rated on a Likert scale ranging from 1 (“never”) to 5 (“always”). An initial set of items was conceptually developed to cover the social interest/drive and affiliation components of social motivation, and the constructs of expressive social communication and social recognition. Out of 58 items, 31 items were hypothesized to tap into social drive/interest and affiliative behaviors, 14 into expressive social communication skills, and 13 into social recognition. Upon the final review of the items, 6 items from the social recognition and 2 from the social drive/interest pools were excluded due to low relevance to the hypothesized constructs. Twelve items are reverse coded so that for all items higher score implies higher endorsement/frequency of a particular behavior. The mean reading level across items is grade 8.7. During initial stages of the survey, parents were able to provide feedback on the questionnaire by indicating whether they perceived questions as meaningful (on a scale from 1 to 4) and whether any of the items were unclear or difficult to understand. Ninety-seven percent of parents indicated that they considered questions as moderately to highly meaningful (22.3% endorsed a rating of 3 and 75.5% a rating of 4) and only 2.2% rated questions as somewhat meaningful. None of the parents endorsed a rating of 1 (not meaningful). Eighty-eight percent of parents reported no issues with item clarity. Out of 12% of parents that suggested certain items were unclear or difficult to understand, only 3 parents endorsed the same item (item 20). Therefore all items were retained for the full survey and the analysis.

*The Social Responsiveness Scale-Second Edition* (*SRS-2* [37])*.* The SRS-2 is a 65-item measure designed to index autism trait severity. The parent report form was used. The following five theoretically derived scales are described in the SRS manual: Social Awareness, Social Cognition, Social Communication, Social Motivation, and Autistic Mannerisms; however, subsequent factorial work has not provided strong support for these subscales [59, 60, 61] suggesting that the SRS might be best conceptualized as a unidimensional measure. Relatively recent factor analysis by Frazier et al. [40] has suggested that unidimensional structure provided a poor fit and that two (Social Communication/Interaction and Restricted/Repetitive Behavior) and five-factor solutions (Social Avoidance, Emotion Recognition, Interpersonal Relatedness, Insistence on Sameness, and Repetitive Mannerisms) might be more optimal. Although 5-factor solution by Frazier and colleagues had strong fit indices, the correlations among derived factors were nevertheless very high. Therefore, in this investigation we have focused on the total SRS score and on the SCI and the RRB scales from the two-factor solution (given that these scales correspond to DSM-5 ASD symptom domains).

*The Child Behavior Checklist, Ages 1.5–5 and 6-18 (CBCL* *[*54*]).* The CBCL is a parent-report instrument designed to assess behavioral and emotional problems in children. It provides eight empirically based syndrome scales that are grouped into internalizing and externalizing problems domains used here. In addition, the CBCL dysregulated profile (CBCL-DP), which is calculated from the anxious/depressed, attention, and aggressive behaviors scales, was used as an index of impairments in self-regulation.

Sample 2

The second sample consisted of parents who participated in an online survey through Survey Sampling International (Shelton, CT). In addition to the SSDS and the SRS-2, the following measures were collected:

*The Strengths and Difficulties Questionnaire (SDQ* [58]*)* is a 25-item parent-report measure of emotional and behavioral problems in children. It provides a total score as well as scores for emotional, conduct, hyperactivity, and peer problems and for prosocial behaviors. The total score of 17 and above indicates clinically significant problems.

*The Need to Belong Scale (NTBS* [55]*)* is a 10-item questionnaire designed to measure a desire for social contact, in particular, affiliation motivation.

*The Child Social Preference Scale (CSPS* [12]*)* is a 15-item questionnaire measure developed to assess the different components of children's social withdrawal. In this study, we focused on the unsociability subscale which assesses the lack of desire and interest to engage in social interactions.

#### Procedures

##### Sample 1

This study was conducted at Stanford University through the Autism and Developmental Disabilities Research Program (ADDRP) in the Department of Psychiatry and Behavioral Sciences. Parents and/or legal guardians participating in the study provided consent and completed all study questionnaires through a secure online portal. Data was managed using Research Electronic Data Capture (REDCap [62]). Licensing permission was obtained from WPS and ASEBA for use of the SRS-2 and CBCL in the online survey. For those families participating in other research studies at Stanford, paper copies of the survey materials were offered if preferred. In addition, parents provided consent for use of data from other studies, including IQ and diagnostic confirmation data.

##### Sample 2

Participants were recruited through Survey Sampling International (Shelton, CT), which specializes in recruiting demographically representative samples for scientific research in the USA. Parents were sent a link to a Qualtrics survey containing consent and questionnaires. Both studies were approved by the Stanford University Institutional Review Board.

### Analysis plan

Prior to running analyses, all questionnaires were screened for missing data. The SSDS was examined for latent components using the Exploratory Structural Equation Modeling (ESEM) framework [63]. ESEM offers considerable advantages over the classic confirmatory factor analysis (CFA) and exploratory factor analysis (EFA) approaches (for a detailed overview see [63, 64]). More specifically, while CFA represents a significant methodological advancement over EFA (e.g., providing a comprehensive set of goodness-of-fit indices, different models estimation), it only allows items to load onto the hypothesized factors, while loadings onto other factors are typically set to 0. Constraining item loading onto only one factor has been highlighted as overly restrictive and unrealistic when applied in psychological research, where items are to be expected to also load onto the non-target factors (constructs) [65]. In the cases where items do indeed show a degree of cross-loading but are artificially set to 0, as is the case in CFA, simulation studies have shown that this results in biased parameter estimates and poor overall fit [64, 66, 67]. Unlike CFA, EFA freely estimates cross-loading of items across all factors; however, it does not provide other benefits related to using CFA [63, 64, 68]. Therefore, we utilized ESEM, a newly developed analytic framework that combines the advantages of both less restrictive approaches such as EFA (e.g., allows item cross-loading) and those of the more advanced approaches such as CFA, in particular, providing goodness-of-fit indices [63]. For confirming the factor structure derived in sample 2, we utilized a confirmatory approach of the ESEM with a target rotation [59 ,62].

ESEM was conducted with MPLUS 8.0 [69]. A maximum likelihood estimator (MLR) was used given that it is robust to non-normal data distribution and appropriate when five or more response categories are used [70]. For consistency, models were also run using the variance-adjusted weighted least squares (WLSMV) estimator. In ESEM, items load onto the main factor and are aimed, but not forced, to load as close to 0 as possible onto other factors. Geomin rotation was used [63]. Only items with loading > .32 were included in the final factor solution [71]. Model fit was evaluated using the following recommended fit indices: the Comparative Fit Index (CFI), the Tucker-Lewis Index (TLI), the Root Mean Square Error of Approximation (RMSEA), and the Standardized Root Mean Square Residual (SRMR). The following cut-offs across the fit indices were applied: (1) CFI and TLI values > .90 indicate adequate fit and > .95 excellent fit; (2) RMSEA < .08 indicates adequate fit and < .06 excellent fit, with 90% confidence intervals required not to cross the .08 boundary and the close fit test to have a *p* value > .05; (3) SRMR < .08. The chi-square index was not used given that it tends to be oversensitive to sample size.

The reliability and construct validity of extracted factors was determined by using the Composite Reliability Index (CR) and examining the strength of the item-item and item-factor correlations. CR was chosen over Cronbach’s alpha as it has been suggested that Cronbach’s alpha underestimates scale reliability in cases when measurement errors are uncorrelated, and in cases when measurement errors are correlated, it can either over- or under-estimate the scale reliability [72, 73]. Convergent and divergent validity were examined by exploring the relationship between SSDS factors with the SRS-2 and CBCL subscales. Relationships with age, sex, and verbal and non-verbal IQ were also explored. In sample 2, relationships between SSDS factors with the CSPS, NTBS, and social avoidance SRS-2 subscale were explored. All correlations were performed through bootstrapping using 5000 resamples to provide more robust statistics and account for the potential skewness of the data [74, 75]. Finally, in Sample 2, group differences in SSDS factor scores between typically developing, children with ASD and children with other clinical conditions were explored using ANOVA. All comparisons were supplemented with the effect sizes. All comparisons, convergent, and divergent validity analyses were run using subscale rather than factor scores.

## Results

### Sample 1

#### Exploratory Structural Equation Modeling (ESEM)

**Fig. 1:**
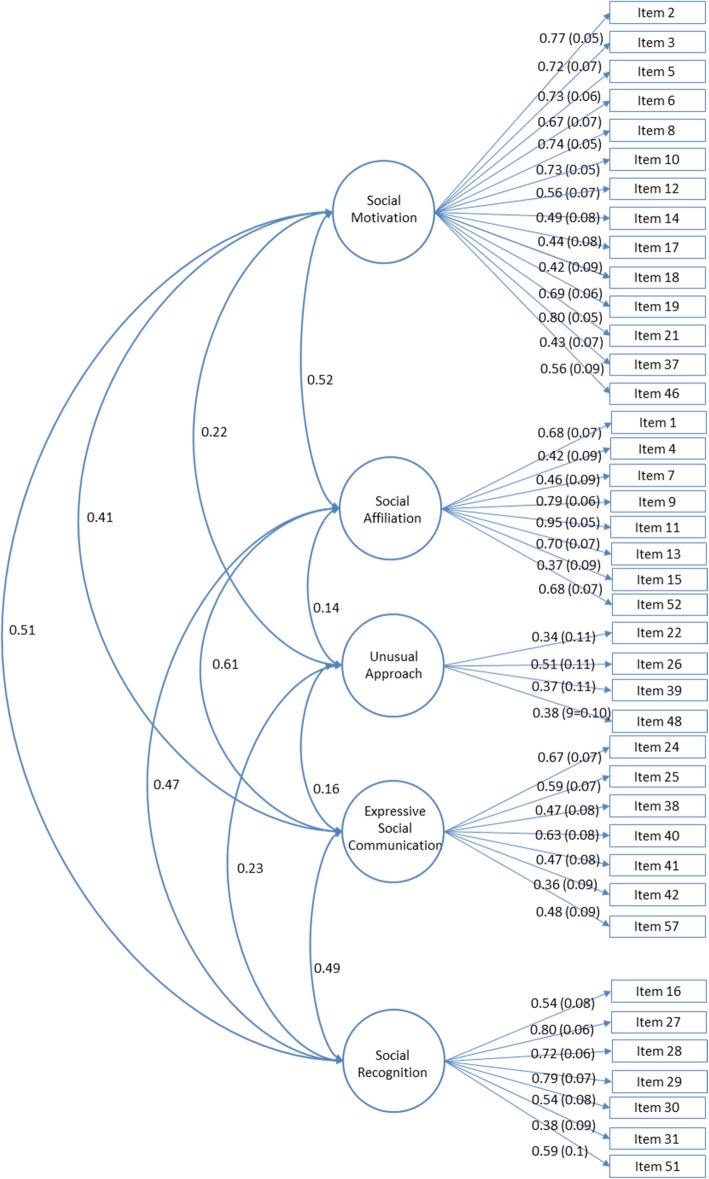
Exploratory Structural Equation Modeling correlated 5-factor solution. Solid lines represent factor loadings and curved lines represent the correlation among factors.

Six ESEM models were run specifying 1-6 factor solutions. Table 2 presents the full list of fit indicators for each of the models. A five-factor model provided adequate to excellent fit to the data as indicated by (1) CFI and TLI values of .940 and .919, respectively, indicating adequate fit; (2) RMSEA = .048 (90% CI, .039, .056) and close fit test *p* = .425, indicating excellent fit; and (3) SRMR = .038 indicating excellent fit. The 5-factor model showed superior fit when compared to 1- to 4-factor solutions (Table 2). Although the 6-factor solution provided a marginally better fit in terms of CFI and TLI, BIC was higher, and the 5-factor model was more parsimonious; therefore, the 5-factor model was retained. The five factors derived through ESEM were interpreted as (1) Social Motivation (example items, “In a social situation, attempts to play with other children instead of avoiding the group”, “Prefers to play with children rather than alone”), (2) Social Affiliation (example items, “Will try to get my attention or interact with me, without being reminded to do so,” “When enjoying something, he/she tries to share that enjoyment with me,” and “Points to objects of interest to share his/her enjoyment with others”), (3) Expressive Social Communication (example items, “Vocalizes and makes eye contact with me when he/she makes a request” and “When a familiar person tries to engage with my child, she/he responds positively and appropriately by smiling, saying hello etc”); (4) Social Recognition (example items, “Understands complex nonverbal gestures used by another person” and “Reads subtle emotions [ex: ashamed, jealous, pleased] in others through their facial expressions”), and (5) Unusual Approach (example items, “Begins interactions/conversations in ways that seem unusual to others” and “Has trouble understanding personal space (e.g., stands too close to others when interacting)”) constructs. Individual factor loadings and correlations among identified SSDS factors are presented in Fig. 1. Given the relatively small sample size, in order to ensure that 5-factor structure was not a result of overfitting, we also considered a 4-factor solution. The identified factors largely matched the SR, ESC and UA factors, and an additional factor encompassed SM and SA factors. Given that other factors were consistent, and that the 5-factor solution provided additional differentiation between SM and SA, the 5-factor was chosen over the 4-factor solution. Analyses were re-run using the WLSMV estimator yielding identical factor composition. Researchers interested in obtaining the full SSDS should contact AYH and JMP.

**Table 2:** Summary of goodness of fit statistics across all tested models

Model	*χ*^2^ (df)	CFI	TLI	RMSEA (90%CI)	SRMR	AIC	BIC	corBIC
ESEM 1 Factor	1938.848** (798)	.688	.664	. 098** (.092; .103)	.105	15676.690	16119.254	15654.027
ESEM 2 Factors	1494.871** (757)	.798	.771	.081** (.075; .087)	.079	15314.712	15880.712	15285.728
ESEM 3 Factors	1191.583** (717)	.870	.844	.066** (.060; .073)	.058	15091.425	15777.850	15056.274
ESEM 4 Factors	977.231** (678)	.918	.896	.054* (.047; .062)	.046	14955.073	15758.913	14913.909
ESEM 5 Factors	859.313** (640)	.940	.919	.048 (.039; .056)	.038	14913.154	15831.398	14866.131
ESEM 6 Factors	968.449** (679)	.948	.926	.046 (.037; .054)	.036	14921.699	15951.337	14868.972

*< .01

**< .001

*AIC* Akaike Information Criterion, *BIC* Bayesian Information Criterion, *corBIC* Sample size adjusted BIC, *ESEM* Exploratory Structural Equation Modeling, *CFI* Comparative Fit Index, *RMSEA* Root Mean Square Error of Approximation, *SRMR* Standardized Root Mean Square Residual, *TLI* Tucker-Lewis Index

#### Reliability and construct validity

The reliability of the derived factors was in the good to excellent range as indicated by Composite Reliability Index scores of .90, .80, .74, .85, and .72 for Social Motivation (SM), Social Affiliation (SA), Expressive Social Communication (ESC), Social Recognition (SR), and Unusual Approach (UA) factors, respectively. Item-subscale correlations are presented in Table 2. The mean correlation of items belonging to a specific subscale was significantly higher with the hypothesized subscale than with other four subscales (mean SM items-SM subscale *r* = .70 [SD = .06], mean SA items-SA subscale *r* = .79 [SD = .06], mean SEC items-SEC subscale *r* = .65 [SD = .08], mean SR items-SR subscale *r* = .73 [*SD* = .04], mean UA items-UA subscale *r* = .69 [*SD* = .12]). See Table 3 for the full detail of mean item-subscale correlations.

**Table 3:** Item-subscale correlations

	Item-subscale correlations				
	SM	SA	ESC	SR	UA
Factor 1: Social Motivation (SM)					
Item 2	.71**	.37**	.23**	.30**	.08
Item 3	.64**	.22*	.26**	.08	.08
Item 5	.77**	.44**	.31**	.34**	.09
Item 6	.71**	.22*	.20*	.29**	.14
Item 8	.76**	.41**	.18*	.46**	.11
Item 10	.80**	.43**	.28**	.39**	.14
Item 12	.72**	.42**	.17	.41**	.07
Item 14	.66**	.51**	.54**	.35**	.03
Item 17	.66**	.38**	.29**	.45**	.25**
Item 18	.62**	.36**	.36**	.38**	.28**
Item 19	.73**	.40**	.24**	.33**	.19*
Item 21	.79**	.47**	.31**	.42**	.18*
Item 37	.63**	.47**	.46**	.58**	.14
Item 46	.63**	.25**	.28**	.12	.17
Mean (SD) item-subscale relationship	.70(.06)	.38(.09)	.29(.10)	.35(.13)	.14(.07)
Factor 2: Social Affiliation (SA)					
Item 1	.50**	.85**	.50**	.34**	.09
Item 4	.46**	.70**	.42**	.32**	.12
Item 7	.36**	.76**	.58**	.23**	.09
Item 9	.32**	.83**	.38**	.34**	.04
Item 11	.42**	.84**	.35**	.35**	.09
Item 13	.46**	.84**	.53**	.44**	.08
Item 15	.32**	.71**	.65**	.29**	.03
Item 52	.45**	.77**	.42**	.40**	.08
Mean (SD) item-subscale relationship	.41(.07)	.79(.06)	.48(.10)	.34(.06)	.08(.03)
Factor 3: Expressive Social Communication (ESC)					
Item 24	.11	.33**	.71**	.25**	.13
Item 25	.20*	.37**	.72**	.30**	.08
Item 38	.37**	.41**	.68**	.36**	.09
Item 40	.13	.34**	.67**	.25**	.15
Item 41	.35**	.35**	.72**	.29**	.05
Item 42	.12	.34**	.49**	.10	.05
Item 57	.28**	.41**	.62**	.19*	.06
Mean (SD) item-subscale relationship	.25(.13)	.39(.07)	.65(.08)	.29(.13)	.10(.04)
Factor 4: Social Recognition (SR)					
Item 16	.48**	.42**	.36**	.69**	.14
Item 27	.30**	.30**	.36**	.78**	.13
Item 28	.42**	.27**	.24**	.73**	.17
Item 29	.28**	.23**	.25**	.77**	.25**
Item 30	.39**	.40**	.43**	.74**	.07
Item 31	.36**	.44**	.50**	.67**	.12
Item 51	.37**	.35**	.40**	.74**	.30**
Mean (SD) item-subscale relationship	.37(.07)	.34(.08)	.36(.09)	.73(.04)	.17(.08)
Factor 5: Unusual Approach (UA)					
Item 22	.18*	.18*	.22*	.17	.80**
Item 26	.28**	.21*	.30**	.26**	.78**
Item 39	− .05	− .03	− .04	.12	.66**
Item 48	.11	− .06	− .07	− .03	.54**
Mean (SD) item-subscale relationship	.13(.14)	.09(.12)	.10(.18)	.13(.12)	.69(.12)

*p < .05

**p < .01

The Social Motivation subscale was strongly associated with the Social Affiliation (*r* = .52, *p* < .001) and the Social Recognition (*r* = .51, *p* < .001) subscales, moderately with the Expressive Social Communication subscale (*r* = .41, *p* < .001) and weakly with the Unusual Approach subscale (*r* = .22, *p* = .006). The Social Affiliation subscale was strongly associated with Expressive Social Communication (*r* = .61, *p* < .001) and moderately with the Social Recognition subscales (*r* = .44, *p* < .001), which, in turn, were moderately correlated with one another (*r* = .47, *p* < .001). The Unusual Approach subscale showed weak association with the Social Motivation (*r* = .22, *p* = .006) and the Social Recognition subscales (*r* = .23, *p* = .004), but not with the Social Affiliation and the Expressive Social Communication (*r* = .14 and .16, respectively) subscales.

#### Validity

A full list of correlations is presented in Table 4. Age was only (positively) correlated with higher Social Recognition subscale scores. Females had significantly higher Social Motivation (*F* = 4.24, *p* = .041, *ƞ*2 *=* .025) and Expressive Social Communication (*F* = 5.15, *p* = .025, *ƞ*2 *=* .03) scores. While Social Motivation, Social Affiliation, and Social Recognition subscale scores were positively associated with both VIQ (*r* = .38, .39, and .39, all *p* < .001, respectively) and NVIQ (*r* = .40, .41, and .34, all *p* < .001, respectively), Expressive Social Communication was not. The Unusual Approach subscale was significantly associated with NVIQ (*r* = 24, all *p* = .033).

**Table 4:** Convergent and divergent validity of the Stanford Social Dimensions Scale

	CA	VIQ^1^	NVIQ^1^	CBCL Int^2^	CBCL Ext^2^	CBCL DP^2^	SRS-2 SCI Raw	SRS-2 SCI *T*	SRS-2 RRB Raw	SRS-2 RRB *T*	SRS-2 Total Raw	SRS-2 Total *T*
Social Motivation	.08	.38**	.40**	− .24**	− .03	− .07	− .61**	− .30**	− .34**	− .35**	− .57**	− .51**
Social Affiliation	.03	.39**	.41**	− .19**	− .08	− .10	− .47**	− .21**	− .27**	− .29**	− .45**	− .40**
Expressive Social Communication	− .03	.12	.10	− .36**	− .23**	− .27**	− .46**	− .22**	− .28**	− .29**	− .44**	− .43**
Social Recognition	.18*	.39**	.34**	− .13	− .10	− .05	− .49**	− .27**	− .25*	− .28**	− .46**	− .40**
Unusual Approach	− .05	.18	.24*	− .21**	− .20*	− .26**	− .43**	− .26**	− .51**	− .49**	− .47**	− .47**

*p < .05

**p < .01

^1^Data available for *N* = 79 individuals

^2^Data available for *N* = 156 individuals

*CA* Chronological Age, *CBCL* Child Behavior Checklist, *DP* dysregulated profile, *Ext* externalizing, *Int* internalizing, *NVIQ* non-verbal IQ, *RRB* restricted/repetitive behavior, *SCI* social communication/interaction, *SRS-2* Social Responsiveness Scale, *VIQ* verbal IQ

The Social Motivation, Social Affiliation, Expressive Social Communication, and Social Recognition subscales were more strongly associated with the SRS-2 social communication/interaction scale (SCI) than with the SRS-2 restricted/repetitive behavior (RRB) scale. The Unusual Approach subscale was more strongly associated with the SRS-2 RRB scale than with SRS-2 SCI scale scores (*r* = − .43 vs − .51); however, this difference did not reach statistical significance. SRS-2 SCI and RRB scores were strongly inter-related (*r* = .75, *p* < .001).

Higher social motivation, social affiliation, and better social skills, as indexed by the higher scores on the Social Motivation, Social Affiliation, Expressive Social Communication, and Unusual Approach subscales, were associated with lower CBCL internalizing problems (*r* = − .24, *p* = .003; *r* = .19, *p* = .016; *r* = − .36, *p* < .001; and *r* = − .21, *p* = .009, respectively). Higher Expressive Social Communication and Unusual Approach subscales were associated with lower CBCL externalizing problems (*r* = − .23, *p* = .004; *r* = − .20, *p* = .011) and with more severe impairments in self-regulation as indexed by the CBCL Dysregulated Profile (*r* = − .27, *p* = .001 and *r* = − .26, *p* = .002).

### Sample 2

#### ESEM

Confirmatory application of ESEM was conducted to confirm the SSDS factors structure identified in sample 1 (ASD sample) in the heterogeneous sample 2 spanning both typical and atypical development. The five-factor model was replicated providing good to excellent fit to the data as indicated by (1) CFI and TLI values of .946 and .931; (2) RMSEA = .044 (90% CI, .041, .048) and close fit test *p* = .997; and (3) SRMR = .026.

#### Reliability and validity

The reliability of the derived factors was in the good to excellent range as indicated by Composite Reliability Index scores of .91, .85, .88, .90, and .73 for Social Motivation (SM), Social Affiliation (SA), Expressive Social Communication (ESC), Social Recognition (SR) and Unusual Approach (UA) factors, respectively. Females had significantly higher Social Motivation (*F* = 8.87, *p* = .015, *ƞ*2 *=* .015) and Expressive Social Communication (*F* = 6.94, *p* = .025, *ƞ*2 *=* .011) scores. The SM subscale was significantly associated with NTBS (*r* = .24, *p* < .001), CSPS Unsociability (*r* = − .52, *p* < .001), and SRS-2 Social Avoidance (*r* = − .67, *p* < .001) scores. The SA subscale was significantly associated with NTBS (*r* = .31, *p* < .001), CSPS Unsociability (*r* = − .24, *p* < .001), and the SRS-2 Social Avoidance factor (*r* = − .31, *p* < .001) scores. SRS-2 Social Avoidance was not significantly associated with NTBS score (*r* = − .05*, p* = .22) and although it was associated with the CSPS Unsociability (*r* = .41, *p* < .001) score, the strength of this association was significantly lower when compared to the SSDS SM-CSPS unsociability association (Fisher *r*-to-*z Z* = 2.62, *p* = .008).

There were significant group differences between TD, ASD, and other clinical conditions groups across all SSDS factors (SM: *F* = 104.22, *p* < .001, *ƞ2 =* .26; SA: *F =* 23.87, *p* < .001, *ƞ2 =* .07; ESC: *F* = 35.59, *p* < .001, *ƞ2 =* .11; SR: *F =* 50.14, *p* < .001, *ƞ2 =* .14; UA: *F =* 61.16, *p* < .001, *ƞ2 =* .17). Posthoc comparisons demonstrated that the TD group had significantly higher SM, SA, ESC, SR, and UA scores (better skills/less impairments) than both ASD and other clinical condition groups, in turn, other clinical condition group had significantly less impairments than the ASD group across all SSDS scales (distribution of SM, SA, ESC, SR, and UA SSDS scores and summary of post hoc comparisons are presented in Additional file 1: Table S2).

## Discussion

The aim of this paper was to provide an initial validation of a multidimensional scale that examines social motivation as well as other key social domains. This newly developed instrument, the Stanford Social Dimensions Scale (SSDS), was designed to comprehensively and sensitively capture individual variation in the social drive/interest and affiliation components of social motivation as well as additional expressive social communication and social recognition dimensions of social functioning. We first aimed to evaluate the factor structure of the SSDS in a sample of individuals with ASD, establish its psychometric properties, and explore the association between the derived factors with age, sex, SRS-2 social communication/interaction (SCI) and the restricted/repetitive behavior (RRB) scale scores, and CBCL externalizing, internalizing and Dysregulated Profile (CBCL-DP) scale scores. Following this, we aimed to (1) confirm the derived SSDS factor structure in a larger, heterogonous sample comprising typically developing children and children with a range of neurodevelopmental and neuropsychiatric disorders, (2) further explore its convergent validity by examining the associations with two well-established measures capturing different aspects of social motivation, and (3) to explore discriminant validity.

Higher social motivation and higher social communication skills (as indexed by the Expressive Social Communication factor), were related to female sex, which is in line with the previous findings on sex differences in this domain in both normative [76] and ASD samples [77, 78]. Better social recognition skills were associated with older age, in line with findings showing that the recognition and interpretation of socially relevant information becomes progressively more sophisticated across development [79, 80]. There were significantly stronger associations between Social Motivation, Social Affiliation, Expressive Social Communication, and Social Recognition subscale scores and the SRS-2 social communication/interaction scale (SCI) as compared to the restricted/repetitive behavior (RRB) scale, indicating good convergent and divergent validity for these factors. There was a stronger (albeit not significant) association between the Unusual Approach factor and the SRS-2 RRB scale, which can be explained by the fact that this factor contains items that capture behaviors that are perceived as unusual in terms of intensity and content, including social initiations and approaches revolving around one’s unusual/intense interests and routines. Importantly, while association between all SSDS subscales (apart from Unusual Approach) were significantly stronger with SRS-2 SCI than with SRS-2 RRB scale scores, SRS-2 SCI and RRB scores were strongly inter-related (*r* = .75, *p* < .001). Higher social motivation, affiliation, and recognition skills were associated with higher cognitive functioning. It will be important to further explore the directionality of these effects in a longitudinal sample. For example, it has been suggested that children with lower levels of social motivation, due to lack of engagement, are exposed less to learning opportunities which can negatively impact cognitive development. Conversely, children with lower levels of cognitive functioning might be rated as lower on social motivation given a more limited range of skills for initiating social engagement. Positive association between social recognition and cognitive functioning is in line with the literature across both normative and atypical development demonstrating the gradual development of social cognition [48, 49]. Lack of an association between cognitive ability and the Expressive Social Communication factor can be explained by the fact that the majority of the items within this factor have been designed to tap into behaviors that are not dependent on verbal ability or cognitive level (e.g., “Will orient toward me when interacting with me” and “When someone smiles at my child he/she will smile back”).

Our finding of an association between the Unusual Approach and the Expressive Social Communication scores and the CBCL externalizing problems scale and poorer self-regulation scores are in line with findings from both ASD and general literature. In both non-ASD and ASD samples, unusual social approach behaviors have been suggested to be related to impairments in self-regulation and externalizing behaviors, rather than to cognitive level, which is in line with our findings that the Unusual Approach factor was associated with CBCL externalizing and Dysregulated Profile scores. For example, both Bonde [81] and Scheeran et al. [82] found that individuals exhibiting active social approach that is inappropriate to a given context (assessed by the Wing Subgroups Questionnaire [83]) exhibited significant impairments in self-regulation and elevated externalizing and internalizing symptoms [82]. Furthermore, a number of studies in non-ASD populations have indicated that children high in externalizing problems exhibit social approach that is characterized either by high intensity or a lack in reciprocity (e.g., to only get their own needs met) and is considered unusual by others [84–86]. Poorer social skills have been associated with internalizing problems (both generalized and social anxiety) in both ASD [87–90] and non-ASD populations [91–93]. Finally, the finding that reduced social approach and affiliative behaviors, as indexed by the Social Motivation and Social Affiliation subscales was associated with higher internalizing CBCL scores is consistent with the general literature on the relationship between lower approach and higher avoidance with higher anxiety levels [95, 96].

Given the focus of the SSDS on assessing different aspects of the social motivation construct, we further explored its association with the Need to Belong Scale (NTBS [55]) and the Child Social Preference Scale (CSPS [12]), designed to measure affiliative and social interest/drive components of social motivation, respectively. The pattern of correlations was supportive of the convergent and divergent validity of the Social Motivation and Social Affiliation subscales of the SSDS given that Social Motivation, which taps into social interest/drive, was associated more strongly with the CSPS unsociability scale which specifically measures social drive, and Social Affiliation was more strongly associated with the NTBS scale. Importantly, the SRS-2 Social Avoidance factor score, which has been suggested to tap into social motivation [40], was not associated with NTBS score, and although it showed significant association with CSPS, the strength of this association was significantly weaker than the association between the SSDS Social Motivation subscale and CSPS as evidenced by the Fisher *r*-to-*z* transformation.

Although this study provides evidence for excellent fit of the five derived factors of the SSDS, good to excellent reliability, strong construct validity and evidence for convergent and divergent validity across two independent samples, further research and scale development are needed. Although the factor structure showed good to excellent fit in independent ASD and heterogeneous samples spanning typical and atypical development, further exploration of the performance and generalizability of the SSDS across the whole spectrum of intellectual functioning is needed and it will be important to investigate the invariance between ASD, other clinical groups, and normative sample. However, given the well-established developmental patterns of different facets of social cognition and social skills [97] and sex differences, it will be necessary to establish invariance of the SSDS factors across sex, age, and cognitive/developmental level prior to comparing the invariance across clinical and normative groups. Although sample size utilized in this study enabled initial testing of the SSDS factor structure, given the number of SSDS items, it was not possible to conduct robust invariance testing across the noted factors. This is one of the key directions for the future development and refinement of this measure. Although parental feedback on the questionnaire was collected, parents were not involved in the initial stages of the instrument development. In addition, this study relied on parent report, and it will therefore be important for future studies to explore the correspondence between parental reports with self and other (e.g., teacher) report and clinician observation versions of the SSDS as well as correspondence with objective and performance-based measures. More specifically, it will be important to further establish the validity of the SSDS by employing a multimodal approach and exploring the relationship between specific facets measured by the SSDS with respective established experimental and behavioral paradigms. Given that ASD is a disorder prototypically associated with a range of social deficits, our initial validation sample focused on ASD. As noted, development of the SSDS was informed by a systematic review of the literature on social processes across both normative and atypical development, and as such the facets it covers and assesses are in line with the current dimensional models of psychopathology, most prominently the RDoC. We were also able to replicate the SSDS factor structure in a large, independent sample that included neurotypical children as well as children with ASD, ADHD, internalizing problems, learning/intellectual disabilities, and a range of other neurodevelopmental and neuropsychiatric disorders. Notably, SSDS factors demonstrated good discriminant validity and dimensionality. More specifically, comparison across normative and clinical samples in terms of the distribution of the SSDS subscale scores showed that the ASD sample had significantly higher impairments/lowest skill levels across all subscales when compared to both normative sample of children with other conditions, who, in turn, had significantly more impairments than the normative sample. However, it will be important to further validate the factor structure and performance of the SSDS in a larger well-characterized clinical sample to show the full range of impairments in different aspects of social functioning.

On a more conceptual level, the SSDS social motivation and affiliation subscales cover a range of different facets of social motivation according to the current theoretical conceptualizations of this construct [7, 98], namely social interest, approach, social liking, and affiliation. However, some aspects of social motivation, such as social orienting, as emphasized by Chevallier et al. [7] are not covered in depth by the current version of the SSDS. In addition, the SSDS social recognition factor includes items assessing processing of both simple and complex affective as well as cognitive information; however, it provides only a single score. Future research should attempt to address these limitations and consider inclusion of additional items so that a comprehensive assessment of different social constructs can be adequately evaluated using the different subscales of the SSDS. This work will need to take into account a long-standing debate on how best to conceptualize recognition and cognition. More specifically, some authors have drawn the distinction between processing of low-level and high-level information (e.g., recognition of facial emotion expression vs. theory of mind) and others between affective and cognitive processing. Therefore, social recognition and cognition should be considered as a multidimensional construct to allow the integration of the complex factors encompassing these processes (for detailed overviews please see [48, 49, 52, 99]). Finally, the Unusual Approach factor contains only four items; however, it was consistent across 4- and 5-factor solutions and confirmed in an independent sample, suggesting that even though it had a limited number of items, it was not a consequence of overfitting. However, it will be crucial to further explore whether this factor will remain in larger samples and to enrich it with a wider range of items assessing atypical approach behaviors, especially including behaviors indicative of externalizing challenges. Therefore, further refinement of the existing subscales, as well as an assessment of additional constructs, are warranted.

Despite these noted limitations, the SSDS has the potential of being a reliable measure that provides a comprehensive and quantitative assessment of social motivation and other important social dimensions and is a promising tool for mapping individual variability across key social domains. As noted, ASD is a highly heterogeneous disorder which has largely limited our ability to uncover its underlying etiology and inform treatments. Therefore, the ability to assess variation in different aspects of social drive and affiliation, social recognition and expression, and atypicalities in social initiation and approach that is afforded by the SSDS opens the possibility of identifying subgroups of individuals with ASD who share distinct patterns of strengths and weaknesses across different social processing dimensions. This can be crucial for understanding and identifying risk and protective factors within ASD social phenotypes and informing the development of targeted, individually tailored treatment options. The social motivation might be a particularly good candidate for differentiating behaviorally and biologically defined subgroups within ASD. For instance, although focusing on the interaction style as a subtyping variable rather than on social motivation per se, work by Wing and Gould [47] has identified three subgroups of individuals with ASD that significantly differed in terms of levels of social interest and motivation to engage. More specifically, on one end of the spectrum, Active but Odd subgroup was characterized by active seeking of social interactions (albeit in unusual and often inappropriate way) and on the other end of the spectrum Aloof subgroup was characterized by both a lack of social interest and a lack of responses to social overtures initiated by others. Therefore, SSDS might be a promising instrument for future studies aimed at identifying more phenotypically and biologically homogenous subgroups. Given robust evidence of ASD heritability, it will also be important to explore heritability of distinct social motivation domains. Although measures such as the SRS-2 have been shown to demonstrate good dimensionality and reasonable coverage of social functioning, as noted, they do not provide detailed sampling of social motivation behaviors limiting their utility for exploring this crucial construct. Therefore, the SSDS fills an important gap in the literature by providing comprehensive sampling of social motivation as well as providing coverage of other crucial social domains. Future work will need to further characterize and refine the structure of the SSDS and incorporate it into intervention, neuroimaging, and genetic studies in order to more fully explore its utility and to explore its performance in larger samples with varying levels of cognitive ability and psychopathology.

## Supplementary information

**Additional file 1:** **Table S1.** Sample 2 Participant Characteristics by Diagnostic Group. **Table S2.** Distribution of SSDS Factor Scores by Diagnostic Group.

## Data Availability

The datasets analyzed during the current study are available from the corresponding author on reasonable request.

## Abbreviations

ADI-R: Autism Diagnostic Interview—Revised
ADOS: Autism Diagnostic Observation Schedule
ASD: Autism Spectrum Disorder
CBCL: Child Behavior Checklist
CSPS: Childhood Social Preferences Scale
DP: Dysregulated profile
ESEM: Exploratory Structural Equation Modeling
NTBS: Need to Belong Scale
NVIQ: Non-verbal intelligence quotient
RRB: Restricted/repetitive behavior
SCQ: Social Communication Questionnaire
SCI: Social communication/interaction
SRS-2: Social Responsiveness Scale
SSDS: Stanford Social Dimensions Scale
VIQ: Verbal intelligence quotient
