# Supplementary material for: Development of the Stanford Social Dimensions Scale: initial validation in autism spectrum disorder and in neurotypicals
Source: Mol Autism. 2019 Dec 18;10:48. doi: 10.1186/s13229-019-0298-9 (PMC6921422; doi:10.1186/s13229-019-0298-9)
Supplement: Supplementary file 1 — Additional file 1: Table S1. Sample 2 Participant Characteristics by Diagnostic Group. Table S2. Distribution of SSDS Factor Scores by Diagnostic Group. [file 13229_2019_298_MOESM1_ESM.docx]

Additional file 1: Table S1. Sample 2 Participant Characteristics by Diagnostic Group

|  | **TD^a^**  **Mean (SD)** | **Other Clinical^b^**  **Mean (SD)** | **ASD^c^**  **Mean (SD)** | **Statistics** | **Posthoc** |
| --- | --- | --- | --- | --- | --- |
| **CA** | 11.38 (4.54) | 11.87 (4.20) | 11.64 (5.00) | F= .70, p= .50, *ƞ2=* .002 | NS |
| **Sex (M/F N)** | 210/224 | 86/69 | 24/6 | *χ^2^*= 12.42, *p*= .002, *Phi*= .14 |  |
| **SRS-2 Total** | 49.30 (8.12) | 67.12 (13.17) | 75.48 (8.98) | F= 266.95, p< .001, *ƞ2=* .46 | a< b, c; b < c |
| **SDQ Total** | 6.10 (5.23) | 17.95 (6.56) | 19.68 (5.31) | F= 306.89, p< .001, *ƞ2=* .50 | a< b, c; b ≈ c |

Note: ASD: Autism Spectrum Disorder; CA: Chronological Age; SDQ: Strengths and Difficulties Questionnaire; SRS: Social Responsiveness Scale; TD: Typically Developing;

Additional file 1: Table S2. Distribution of SSDS Factor Scores by Diagnostic Group

|  | **TD^a^**  **Mean (SD)** | **Other Clinical^b^**  **Mean (SD)** | **ASD^c^**  **Mean (SD)** | **Posthoc** |
| --- | --- | --- | --- | --- |
| **SM** | 57.70 (8.27) | 48.05 (9.46) | 41.68 (9.19) | a> b, c; b> c |
| **SA** | 31.15 (5.81) | 28.61 (5.59) | 25.13 (5.08) | a> b, c; b> c |
| **ESC** | 31.07 (4.94) | 28.01 (5.21) | 25.32 (4.53) | a> b, c; b> c |
| **SR** | 29.03 (5.29) | 25.63 (5.22) | 20.90 (5.76) | a> b, c; b> c |
| **UA** | 15.39 (3.32) | 12.49 (3.40) | 10.97 (2.81) | a> b, c; b> c |

Note: ASD: Autism Spectrum Disorder; ESC: Expressive Social Communication; SA: Social Affiliation;

SM: Social Motivation; SR: Social Recognition; TD: Typically Developing; UA: Unusual Approach
